# Fractal analysis of extracellular matrix for observer-independent quantification of intestinal fibrosis in Crohn’s disease

**DOI:** 10.1038/s41598-024-54545-4

**Published:** 2024-02-17

**Authors:** Marie-Christin Weber, Konstantin Schmidt, Annalisa Buck, Atsuko Kasajima, Simon Becker, Chunqiao Li, Stefan Reischl, Dirk Wilhelm, Katja Steiger, Helmut Friess, Philipp-Alexander Neumann

**Affiliations:** 1 Department of Surgery, TUM School of Medicine and Health, Technical University of Munich, Ismaninger Str. 22, 81675 Munich, Germany; 2 Institute for Advanced Study, Technical University of Munich, Munich, Germany; 3 Institute of Pathology, TUM School of Medicine and Health, Technical University of Munich, Munich, Germany; 4 Department of Mathematics, ETH Zurich, Zurich, Switzerland; 5 Institute of Diagnostic and Interventional Radiology, TUM School of Medicine and Health, Technical University of Munich, Munich, Germany

**Keywords:** Crohn’s disease, Intestinal fibrosis, Histology, Fractal dimension analysis, Inflammatory bowel disease, Crohn's disease, Translational research

## Abstract

Prevention of intestinal fibrosis remains an unresolved problem in the treatment of Crohn’s disease (CD), as specific antifibrotic therapies are not yet available. Appropriate analysis of fibrosis severity is essential for assessing the therapeutic efficacy of potential antifibrotic drugs. The aim of this study was to develop an observer-independent method to quantify intestinal fibrosis in surgical specimens from patients with CD using structural analysis of the extracellular matrix (ECM). We performed fractal analysis in fibrotic and control histological sections of patients with surgery for CD (n = 28). To specifically assess the structure of the collagen matrix, polarized light microscopy was used. A score to quantify collagen fiber alignment and the color of the polarized light was established. Fractal dimension as a measure for the structural complexity correlated significantly with the histological fibrosis score whereas lacunarity as a measure for the compactness of the ECM showed a negative correlation. Polarized light microscopy to visualize the collagen network underlined the structural changes in the ECM network in advanced fibrosis. In conclusion, observer-independent quantification of the structural complexity of the ECM by fractal analysis is a suitable method to quantify the degree of intestinal fibrosis in histological samples from patients with CD.

## Introduction

The prevention of intestinal fibrosis formation is still an unsolved problem in the management of Crohn’s disease (CD) as no antifibrotic therapies are available yet^1,2^. In CD, chronic intestinal inflammation leads to fibrotic remodeling of the intestinal wall by the accumulation of extracellular matrix (ECM) as well as smooth muscle hypertrophy resulting in organ dysfunction and bowel strictures^3,4^. Approximately 50% of patients with CD are estimated to require surgical interventions for complications related to intestinal fibrotic strictures at least once in their lifetime^5,6^.

Appropriate imaging techniques including computer tomography and magnetic resonance imaging for the diagnosis of fibrotic intestinal strictures need to be developed and validated to serve as outcome parameters for clinical trials and thus aid the development of antifibrotic agents^7^. Histopathological evaluation of surgical specimens and their correlation to imaging data is used for the validation of imaging techniques for the diagnosis of intestinal fibrosis severity. Various semiquantitative scores and histopathological diagnostic items to grade intestinal fibrosis severity already exist. Existing scoring systems for intestinal fibrosis in CD evaluate the localization and the amount of ECM deposition within the intestinal wall, mainly using semi-quantitative grading of different items. Some scores include criteria such as smooth muscle hypertrophy or hyperplasia, vascularization, or neural hypertrophy and assess the different layers of the intestinal wall separately^4^. However, so far there is no standardized histopathological scoring system that is routinely used as outcome parameter in clinical trials^8,9^.

In other organ systems such as the liver and lung, measuring the amount of ECM and collagen in histological tissue sections can be used as a quantitative observer-independent tool for fibrosis scoring. Due to the multi-layered structure of the intestinal wall, measuring the ECM fraction of the histological section can be biased for example due to other pathological features of CD strictures such as smooth muscle hyperplasia. However, it has been shown that not only do ECM and collagen fibers accumulate during wound healing and the resulting scar formation but the ECM network also increases its structural complexity^10^. Fractal analysis is a method to quantify the complexity of a geometrical pattern and has been applied to many applications in pathology already^11^. Recently, Jiang et al. have applied fractal analysis to quantify structural changes in ECM networks during scar formation^12^. We hypothesized that during the progression of fibrosis, the structural complexity of the ECM increases, and that this phenomenon could be used to quantify intestinal fibrosis in histological samples of patients with CD related intestinal fibrosis.

In this study, we introduce a new histological method to quantify intestinal fibrosis in CD using structural analysis of the ECM and collagen network. Firstly, we used fractal analysis of the ECM in Masson’s trichrome stained histological sections of the fibrotic focus as well as the resection margins of surgical specimens from patients with CD to measure the structural complexity of the ECM as an observer-independent method for fibrosis quantification. Furthermore, we analyzed picrosirius red stained histological sections using polarized light microscopy to specifically visualize the collagen network within the histological sections to confirm the structural differences of the ECM network associated with intestinal fibrosis.

## Materials and methods

### Study design

Histological sections from 28 patients that underwent bowel resection surgery for CD at the Klinikum rechts der Isar, Technical University Munich, were included in this single-center retrospective study. The primary indication for surgery was clinical bowel obstruction due to fibrotic intestinal strictures for 18 (64%) patients and a combination of intestinal fibrosis related strictures and fistulas or abscesses for 10 (36%) patients (Table 1). Histological samples were provided by the tissue bank of the Institute of Pathology, Klinikum rechts der Isar, Technical University of Munich (MTBIO). Informed consent was obtained from each patient before inclusion in the tissue bank of the Institute of Pathology, Klinikum rechts der Isar, Technical University of Munich (MTBIO). Only patients of which representative histological samples from the resection margins and the focus with maximal fibrotic involvement were available, were included in the study. The study was conducted in accordance with the Declaration of Helsinki and was approved by the Ethics Committee of the Faculty of Medicine, Technical University of Munich (651/21 S-NP).

**Table 1:** Patient characteristics.

Cohort [n]	28 [100%]
Age [years] [range; average]	26–77; 46.9
Sex	
Female	12 [57%]
Male	16 [43%]
Disease phenotype	
Stricturing only	18 [64%]
Additional fistulating manifestation (excluding perianal fistulas)	10 [36%]
Prior Crohn’s related bowel resection surgery	13 [46%]
Type of surgery	
Ileocecal resection	16 [57%]
Ileum/colon segment resection	5 [18%]
Anastomosis resection	7 [25%]
Urgency of surgery	
Elective	19 [68%]
Urgent	4 [14%]
Emergency	5 [18%]

### Histology

For each patient, two formalin-fixed paraffin embedded (FFPE) histological samples were analyzed; one from the resection margin and one from the fibrotic focus. Transmural histological sections (3 µm) were stained using the Masson trichrome with Aniline Blue and picrosirius red staining kits (both Morphisto Ltd., Offenbach am Main, Germany) according to the manufacturer’s instructions. Brightfield slide scanning was performed using a high-capacity digital pathology scanner (Aperio AT2, Leica Biosystems, Wetzlar, Germany) at 400 × magnification. QuPath software was used for visualization of scanned images (.svs files)^13^. Image analysis was further performed using FIJI/ImageJ with the FracLac plugin after exporting the whole slide scan as .tif files from QuPath (down sampling factor of 15)^14,15^.

Polarized light microscopy was performed manually on picrosirius red stained sections using a light microscope and two linear polarizing filters (section “Polarized light microscopy (fiber alignment and color score)”; Fig. 2A, Supplementary Fig. 2).

All image analysis was performed blinded for the localization of the sections within the surgical specimen (resection margin vs. fibrotic focus) and other patient data.

### Image analysis

The following parameters were determined during image analysis: fibrosis score, ECM fraction, fractal dimension and lacunarity (as outcome measures of fractal analysis), fiber alignment and fiber color score (as outcome measures of polarized light microscopy). All image analyses were performed on the entire slide.

#### Fibrosis score

As a reference for the newly defined outcome parameters of this study we used an established semiquantitative histopathological fibrosis score adapted from Adler et al.^16^ that was applied to grade the microscopic degree of fibrosis in the histological sections stained with Masson trichrome (Table 2). Fibrosis scoring was performed by a board-certified pathologist.

**Table 2:** Histological fibrosis score.

Fibrosis grade	Histological findings
0	No fibrosis
1	Minimal fibrosis in submucosa or subserosa
2	Increased submucosal fibrosis, septa into muscularis propria
3	Septa through muscularis propria, increase in subserosal collagen
4	Significant transmural scar, marked subserosal collagen

#### ECM fraction

As another reference for the newly defined outcome parameters in this study, we used the proportionate ECM fraction as a quantitative measure for tissue fibrosis^17^. Masson trichrome staining with Aniline Blue results in a blue staining for ECM proteins. We thus used color thresholding to quantify the ECM area fraction from the total tissue area on the histological slides. The proportionate ECM fraction was defined as the ratio of the blue stained area to the total tissue area (Supplementary Fig. 1).

#### Fractal analysis (fractal dimension and lacunarity)

**Figure 1:**
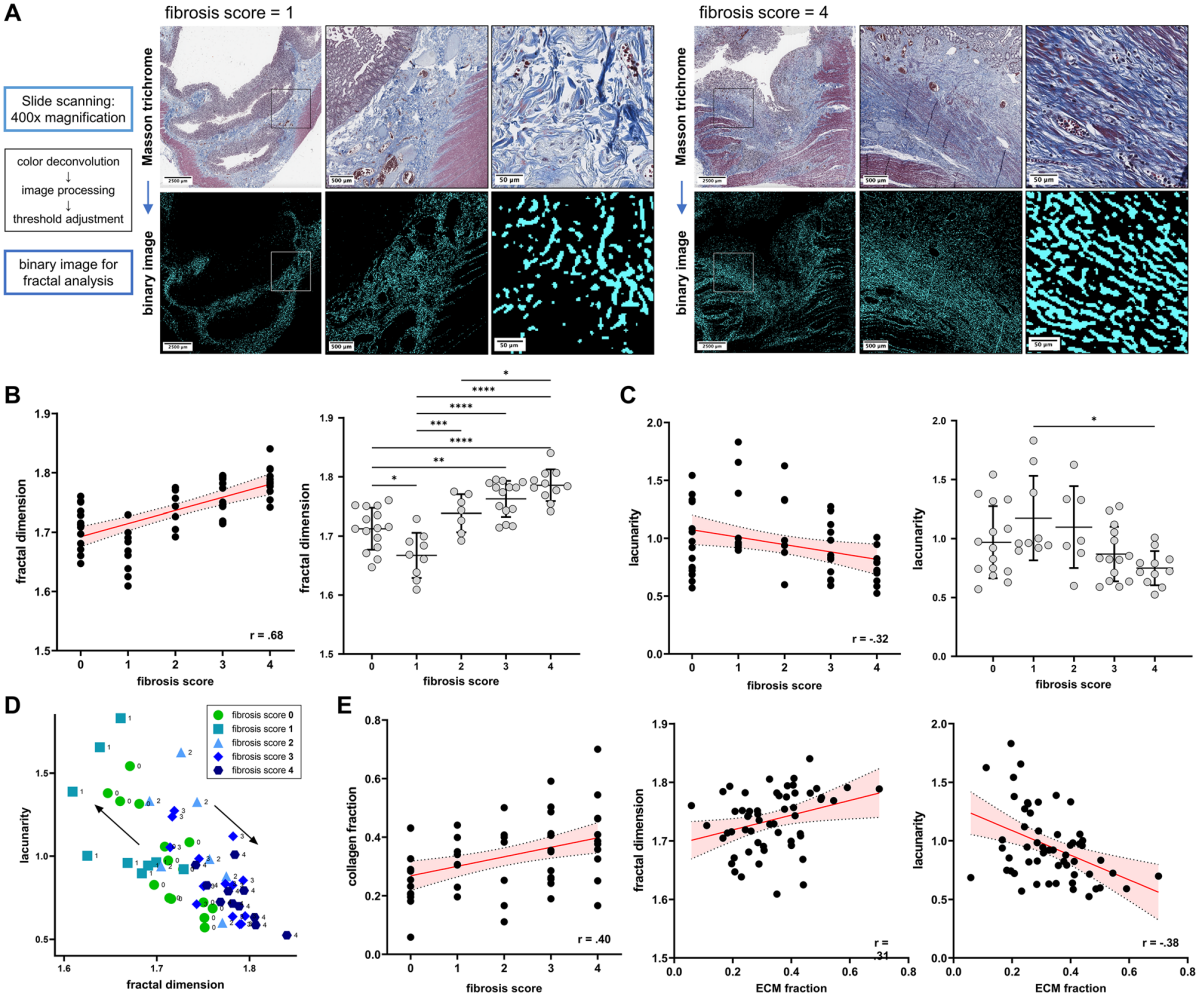
Fractal dimension analysis of extracellular matrix in Masson trichrome stained histological sections. (**A**) Representative images of computational analysis using the FracLac Plugin in FIJI/ImageJ from one patient with a fibrosis score of 1 and one patient with a fibrosis score of 4. (**B**) Spearman correlation analysis (left graph) and one-way ANOVA with Tukey’s multiple comparison test (right graph) of the fractal dimension with the histological fibrosis score. Left graph: data are represented as dots of individual values (xy-graph); red line = best-fit line with 95% confident interval bands. Right graph: data are represented as dots of individual values (column scatter graph), with mean ± SD. (**C**) Spearman correlation analysis (left graph) and one-way ANOVA with Tukey’s multiple comparison test (right graph) of the lacunarity with the histological fibrosis score. Left graph: data are represented as dots of individual values (xy-graph); red line = best-fit line with 95% confident interval bands. Right graph: data are represented as dots of individual values (column scatter graph), with mean ± SD. (**B**,**C**) *p < 0.05, **p < 0.01, ***p < 0.001, ****p < 0.0001. (**D**) Scatter plot of fractal dimension and lacunarity values with color coding for associated fibrosis score. Black arrows demonstrate a trend towards expansion of the matrix (fractal dimension ↓, lacunarity ↑) for samples with early fibrosis (fibrosis score 1) and a contraction of the matrix for samples with advanced fibrosis (fractal dimension ↑, lacunarity ↓) (**E**) Spearman correlation of the fibrosis score with the proportionate extracellular matrix fraction (ECM) fraction as well as correlation of the proportionate (ECM) fraction with fractal dimension and lacunarity values. Data are represented as dots of individual values, (xy-graphs); red lines = best-fit lines with 95% confident interval bands.

Fractal dimension, a measure for the complexity and lacunarity, a measure for the porosity or compactness of the given pattern are the outcome parameters of fractal analysis used in this study. For fractal analysis, overview scans at 400 × magnification from histological sections stained with Masson trichrome with Aniline Blue were used. The method for fractal analysis including standardized image preprocessing was adapted from the established protocol by Jiang et al.^12^. Prior to performing fractal analysis, images were preprocessed in FIJI/ImageJ to enhance the structural characteristics and eliminate interfering artifacts. After converting the image into the CMYK color space, the color channels were split, and the cyan channel was selected for further processing. The background was subtracted (rolling ball radius: 20 pixels), the contrast enhanced (normalized, pixel saturation: 0.1) and the following filters were applied: “unsharp mask” (radius: 2, mask: 0.6) and “median” (radius: 1). The image was then converted into 8-bit and the threshold “Moments” was applied followed by noise reduction using the despeckle option. The FracLac plugin was then applied for fractal analysis using the box counting method with the sliding box scan for fractal dimension and lacunarity calculation (12 grids at default sampling sizes, maximum grid caliber = 45% of image size) (Fig. 1A). The box counting method is a widely used method to determine the fractal dimension as a measure for the geometrical complexity of histological patterns. The algorithm involves overlaying a grid of square boxes with varying sizes onto the pattern, which in this case is the binary image obtained after preprocessing the histological image as described above. By counting the boxes needed to cover the object at each box size, a logarithmic relationship between the box size and the corresponding box count is established of which the fractal dimension can be calculated. The outcome measure of fractal analysis, the fractal dimension, is a unit-free measure. Given, that the dimension of a simple line is 1 and of a filled square is 2, the expected range of the fractal dimension of the pattern analyzed during fractal analysis of histological patterns is between 1 and 2. Further details on the box counting method are outlined in the Supplementary Material 4.

#### Polarized light microscopy (fiber alignment and color score)

**Figure 2:**
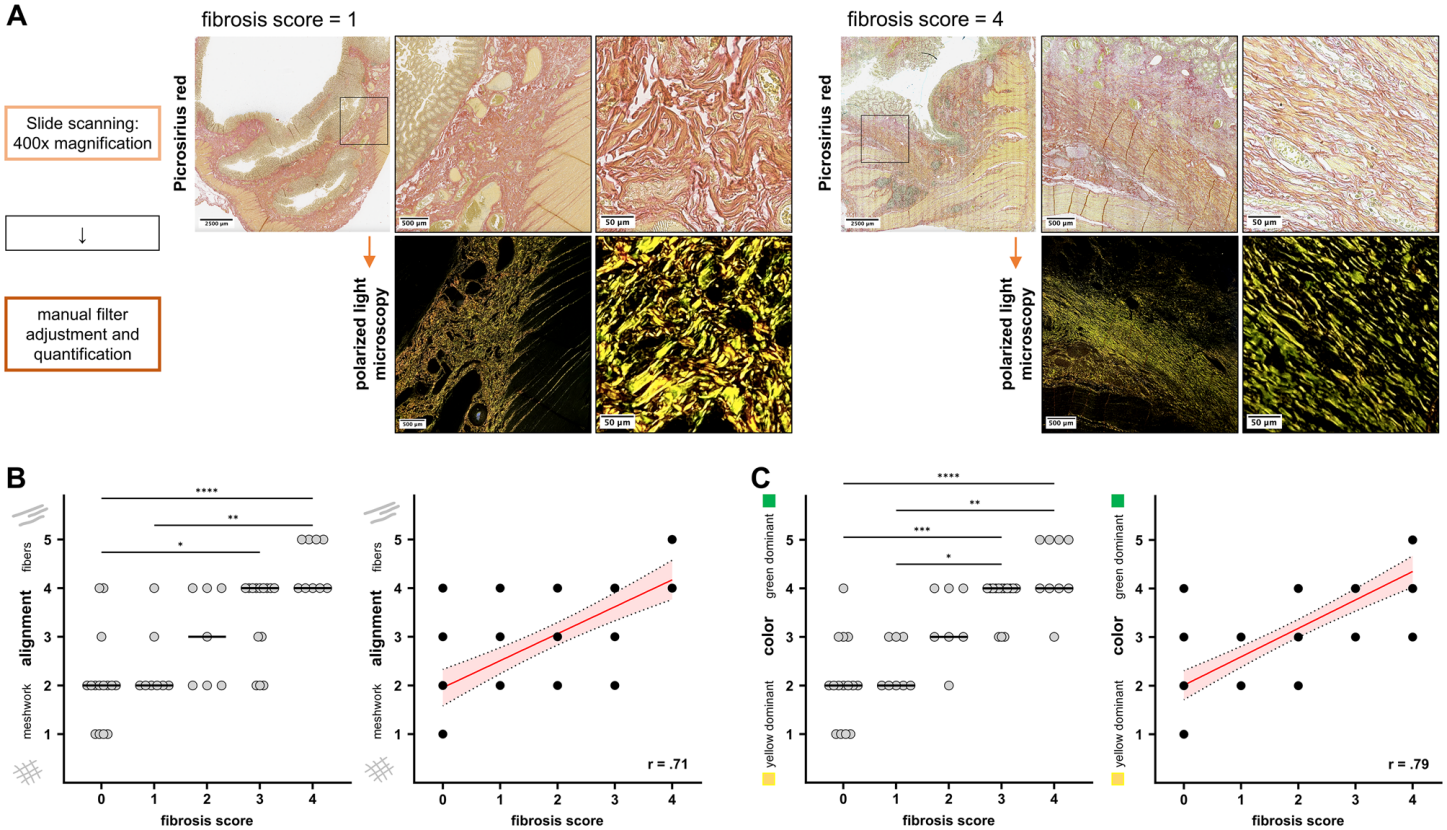
Polarized light microscopy of picrosirius red stained histological sections. (**A**) Representative images of polarized light microscopy ImageJ from one patient with a fibrosis score of 1 and one patient with a fibrosis score of 4. (**B**) Kruskal–Wallis test with Dunn’s correction for multiple comparison testing (left graph) and Spearman correlation analysis of the alignment score with the histological fibrosis score (right graph). Left graph: data are represented as dots of individual values (column scatter graph), black lines represent median alignment score. Right graph: data are represented as dots of individual values (xy-graph); red line = best-fit line with 95% confident interval bands. (**C**) Kruskal–Wallis test with Dunn’s correction for multiple comparison testing (left graph) and Spearman correlation analysis of the color score with the histological fibrosis score (right graph). Left graph: data are represented as dots of individual values (scattered dot-plot), black lines represent median alignment score. Right graph: data are represented as dots of individual values (aligned dot-plot); red line = best-fit line with 95% confident interval bands. (**B**,**C**) *p < 0.05, **p < 0.01, ***p < 0.001, ****p < 0.0001.

As tools to further analyze the structure of the collagen matrix as measures for tissue fibrosis, polarized light microscopy was performed. As outcome parameters of polarized light microscopy, we defined a fiber alignment score ranging from a meshwork-like pattern to parallel fiber alignment as well as a color score ranging from orange-yellow to green (Table 3). For polarized light microscopy, picrosirius red stained histological sections were scored using a light microscope (Olympus BX53, Tokyo, Japan) with two linear polarizing filters. Picrosirius polarized light microscopy allows for selective visualization of fibrillar collagen fibers^18^. The filters were manually adjusted to 90° counterrotation for optimal alignment during scoring, whole slides were analyzed at 40 × to 100 × magnification. Two main structural characteristics of fiber alignment were identified, a meshwork-like pattern and parallel arranged fibers. As the fiber alignment was not homogeneous within the individual histological sections, quantification of the whole slide was performed manually according to a score ranging from 1 (meshwork-like pattern), 2 (predominantly meshwork-like pattern), 3 (mixed pattern), 4 (predominant parallel fiber alignment) to 5 (parallel fiber alignment) (Fig. 2A, Table 3). For quantification of the color in our samples, we manually scored the color of the collagen fibers under polarized light ranging from 1 (orange-yellow dominant) to 5 (green dominant) (Fig. 2A, Supplementary Fig. 2, Table 3). Representative images were digitalized and documented using the CellSense software (Olympus, Tokyo, Japan).

**Table 3:** Fiber alignment and fiber color score (polarized light microscopy, picrosirius red staining).

Fiber alignment score	
Score	Fiber alignment pattern
1	Meshwork-like pattern
2	Predominant meshwork-like pattern
3	Mixed pattern
4	Predominant parallel fiber alignment
5	Parallel fiber alignment

Fiber color score	
Score	Fiber color
1	Orange-yellow dominant
2	Predominant orange-yellow fibers with occasional green fibers
3	Mixed yellow and green fibers
4	Predominant green fibers with occasional orange-yellow fibers
5	Green dominant

### RNA isolation and qPCR

The mRNA was isolated from FFPE tissue sections from the same FFPE blocks that were used for the histology. Similar to the histologic analysis, one sample from the resection margin and one sample from the fibrotic focus were analyzed per patient. The Qiagen RNeasy FFPE Kit (Qiagen, Hilden, Germany) was used for RNA isolation according to the manufacturer’s protocol. First strand cDNA was synthesized using the Qiagen RT^2^ First Strand kit (Qiagen, Hilden, Germany). qPCR was performed on a LightCycler 480 platform (Roche, Basel, Switzerland) using the KAPA SYBR Fast DNA polymerase (Roche, Basel, Switzerland). The following primers were used: *PPIB*: forward 5′-TGTGGTGTTTGGCAAAGTTC-3′, reverse 5′-GTTTATCCCGGCTGTCTGTC-3′; *COL1A1*: forward 5′-GAGGGCCAAGACGAAGACATC-3′, reverse 5′-CAGATCACGTCATCGCACAAC-3′; *COL3A1*: forward 5′-GCTGGCTACTTCTCGCTCTG-3′, reverse 5′-TCCGCATAGGACTGACCAAG-3′. Relative mRNA expression is represented as relative expression (2^−ΔCT^) normalized to *PPIB*.

### Immunofluorescence staining and imaging

FFPE slides were deparaffinized, rehydrated and heat mediated antigen retrieval was performed using citrate buffer, pH 6.0. For quenching of free aldehyde groups, a 50 mM NH4Cl solution was used, followed by blocking with 5% (w/vol) bovine serum albumin for 1 h. Slides were subsequently incubated with the respective first antibody (COL1: ab88147, 1:200 and COL3: ab7778, 1:200, both Abcam, Cambridge, GB) at 4 °C overnight, followed by 1 h incubation at RT with the secondary antibody (ab15007, Alexa Fluor 488 (Abcam, Cambridge, GB), A11005, Alexa Fluor 594 (Invitrogen, Waltham, USA), both diluted 1:300). Slides were then mounted with Fluoroshield (Sigma-Aldrich, St. Louis, USA) and imaged using the Zeiss Axio Observer Z1 microscope (Carl Zeiss AG, Oberkochen, Germany).

### Statistical analysis

Statistical analysis and data visualization were performed using GraphPad Prism (Version 9.3.0, GraphPad Software, San Diego, USA). Data is presented as mean ± SD or SEM as indicated in the figures. Shapiro–Wilk test was used to test for normal distribution of data points. Statistical differences were analyzed using unpaired *t*-test and one-way ANOVA with Tukey’s correction for multiple comparisons for parametric, normally distributed parameters or Mann–Whitney *U* test and Kruskall–Wallis test with Dunn’s correction for multiple comparisons for non-parametric parameters as indicated in the figure legends. Spearman correlation was used to analyze correlation between parameters. A *p-*value < 0.05 was considered as statistically significant.

### Ethical considerations

The study was approved by the Ethics Committee of the Faculty of Medicine, Technical University of Munich (651/21 S-NP). Informed consent was obtained from each patient before inclusion in the tissue bank of the Institute of Pathology, Klinikum rechts der Isar, Technical University of Munich (MTBIO).

## Results

### Fractal dimension and lacunarity correlate with histopathological fibrosis score

The outcome parameters of the fractal analysis are the fractal dimension, a measure for the complexity and lacunarity, a measure for the porosity of the given pattern. We correlated the two parameters (fractal dimension and lacunarity) to an established histopathological fibrosis score (Table 2). We found a positive correlation between the histological fibrosis score and the fractal dimension (r = 0.68, p < 0.0001). The fractal dimension was significantly different between sections with different fibrosis scores. Interestingly, the fractal dimension was lower in sections with a fibrosis score of 1 compared to a fibrosis score of 0 (mean fractal dimension 1.71 ± 0.035 vs. 1.67 ± 0.038, p = 0.016) while it increased again for sections with a fibrosis score of 2 (mean fractal dimension 1.74 ± 0.032), 3 (mean fractal dimension 1.76 ± 0.030) and 4 (mean fractal dimension 1.79 ± 0.027) (Fig. 1B). The lacunarity was negatively correlated to the fibrosis score (r = − 0.32, p = 0.016). Lacunarity was only significantly different between section with a fibrosis score of 1 and 4 (mean lacunarity 1.17 ± 0.36 vs. 0.75 ± 0.15, p = 0.014) and an increase in lacunarity could be seen between sections with a fibrosis score of 0 compared to 1 (Fig. 1C). Values for fractal dimension and lacunarity were themselves negatively correlated (r = − 0.70, *p* < 0.0001). The decrease in fractal dimension and increase in lacunarity in sections with fibrosis score of 1 compared to a fibrosis score of 0 can be interpreted as an expansion of the ECM network during early and contraction towards a more complex structure during late stages of fibrosis development (Fig. 1D).

### Correlation of fractal dimension and lacunarity to ECM fraction as an established histomorphometric parameter

Previous studies have assessed the collagen proportionate area by computer-aided digital image analysis as a parameter to quantify fibrosis in CD^17^ and other organ systems^19,20^. We assessed the proportionate ECM fraction using a color threshold method on Masson trichrome stained histological sections to analyze the correlation between the quantity of collagen with its structural properties (Supplementary Fig. 1). First, we analyzed the association between the ECM fraction and the fibrosis score. There was a positive correlation between the ECM fraction and the fibrosis score (r = 0.40, p = 0.005) (Fig. 1E). Furthermore, the positive correlation between the fibrosis score and the fractal dimension was higher than the correlation between the fibrosis score and the ECM fraction indicating that the structural changes in the ECM network might even be more prominent during fibrosis development than just the excessive accumulation of ECM.

As expected from its positive correlation with the fibrosis score, the ECM fraction was also positively correlated with the fractal dimension (r = 0.31, p = 0.022) and negatively correlated with lacunarity (r = − 0.38, p = 0.004) (Fig. 1E). However, although there was a positive correlation between the ECM fraction and the fibrosis score, unlike the fractal dimension, the ECM fraction was not statistically different between samples with different fibrosis scores (Fig. 1E).

In conclusion, our data indicate that during fibrosis progression, not only the proportionate ECM fraction but also the structural complexity of the ECM as measured by fractal analysis increases.

### Structural analysis of collagen matrix using polarized light microscopy reveals fibrosis related changes in collagen fiber alignment

The combination of picrosirius red staining and polarized light microscopy is a tool for specific visualization of fibrillar collagens in histological sections^21^. Thus, to further analyze the ECM structure, we used this method to visualize the collagen fiber alignment in our histological sections. We found that sections with no to minimal fibrosis showed a meshwork (or lattice) like collagen fiber alignment which is known to be the physiological arrangement of collagen fibers in the intestinal submucosa (Supplementary Fig. 2)^22^.

In sections with a higher fibrosis score, the meshwork-like arrangement of collagen fibers progressed into a parallelization and thickening of collagen fibers (Fig. 2A). The median fiber alignment score (Table 3) was 2 for samples with a fibrosis score of 0 and 4 for samples with a fibrosis score of 4 (*p* < 0.0001). Fiber alignment was positively correlated with the fibrosis score (r = 0.71, *p* < 0.0001) (Fig. 2B).

The median color score (Table 3) was 2 for samples with a fibrosis score of 0 and 4 for samples with a fibrosis score of 4. The color score thus correlated positively with the histological fibrosis score (r = 0.79, *p* < 0.0001) (Fig. 2C).

Taken together, visualization of the collagen structure using picrosirius polarized light microscopy, we could detect structural changes in the collagen fiber alignment during fibrosis progression with a transformation of a meshwork-like pattern towards a parallel alignment of collagen fibers. These structural changes were accompanied by a change in the color of the polarized light (Supplementary Fig. 2). These finding further support the hypothesis that during fibrosis progression, ECM accumulation is accompanied by structural changes in the ECM.

### *COL1A1*/*COL3A1* ratio is increased in fibrotic samples

**Figure 3:**
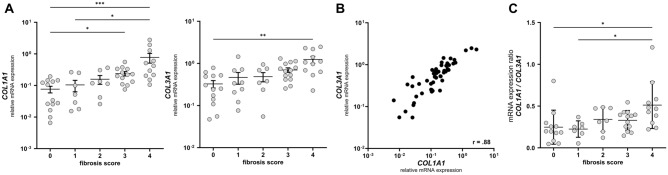
COL1A1 and COL3A1 gene expression. (**A**) Relative mRNA expression of COL1A1 (left) and COL3A1 (right) normalized to PPIB. Data are represented as dots of individual values (column scatter graph), with mean ± SEM. Kruskal–Wallis test with Dunn’s correction for multiple comparison testing. (**B**) Scatter blot demonstrating the correlation between relative COL1A1 and COL3A1 expression. Data are represented as dots of individual values (xy-graph). Spearman correlation analysis. (**C**) Expression ratio of COL1A1 over COL3A1. Data are represented as dots of individual values (column scatter graph), with mean ± SD. Kruskal–Wallis test with Dunn’s correction for multiple comparison testing. (**A**,**C**) *p < 0.05, **p < 0.01, ***p < 0.001.

As some authors suggest that the color of the collagen fibers in picrosirius light microscopy is dependent on the composition of the collagen fibers regarding the distribution of collagen type I and collagen type III, we performed gene expression analysis of *COL1A1* and *COL3A1* from RNA isolated from the corresponding FFPE slides to histological analyses. *COL1A1* and *COL3A1* gene expression was significantly higher in sections with a corresponding fibrosis score of 4 compared to a fibrosis score of 0 (Fig. 3A). Accordingly, *COL1A1* and *COL3A1* gene expression were positively correlated (r = 0.88, *p* < 0.0001) (Fig. 3B). To evaluate whether the expression ratio of both collagens changes during fibrosis progression we calculated the mRNA expression ration of *COL1A1* over *COL3A1*. With an increasing fibrosis score, the expression ratio increased, indicating a predominance of *COL1A1* in samples with a higher fibrosis score (Fig. 3C). Immunofluorescence staining of collagen type I and III showed a higher coverage of collagen type I compared to collagen type III within fibrotic histological sections whereas in sections with no to minimal fibrosis, collagen fibers showed an overlapping signal for both types of collagens (Supplementary Fig. 3).

### Extracellular matrix at the fibrotic focus shows significant structural differences compared to resection margins

**Figure 4:**
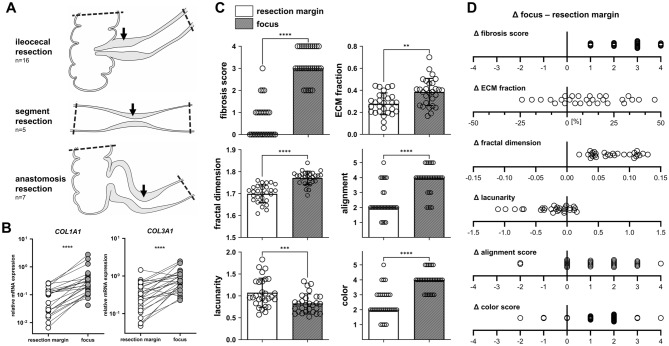
Association of histological fibrosis measurements with localization of the samples within surgical specimen. (**A**) Graphical representation of the localization of the samples. Dashed line = resection margins, black arrow = fibrotic focus. (**B**) Relative mRNA expression of COL1A1 and COL3A1 normalized to PPIB. Data are represented as dots of individual values with connection line for each patient (column scatter graph). Mann–Whitney test, ****p < 0.0001. (**C**) Comparison of different measurements for tissue fibrosis between resections margins and the fibrotic focus. Data are represented as dots of individual values (column scatter graphs) with bar height representing mean ± SD (fractal dimension, lacunarity and extracellular matrix (ECM) fraction) or median (fibrosis, alignment and color score). **p < 0.01, ***p < 0.001, ****p < 0.0001. (**D**) Difference (Δ) of the individual measurements between the fibrotic focus and the resection margins for individual patients. Dots represent Δ for each patient.

In a final step, we compared collagen gene expression, the established histological methods (fibrosis score and ECM fraction) as well as the structural parameters for fibrosis quantification developed in this study between the resection margins and the fibrotic disease focus within the surgical specimen. To do so, for each patient one histological section from the resection margin and one from the fibrotic focus were included in the analysis. Samples from 16 patients that had primary ileocecal resection, 5 patients with segment resections of either small intestine or colon and 7 patients with anastomosis resection due to fibrotic recurrence were included (Fig. 4A). *COL1A1* (mean relative expression 0.45 vs. 0.09, p < 0.0001) and *COL3A1* (mean relative expression 0.37 vs. 0.91, p < 0.0001) mRNA expression was as expected significantly higher within the fibrotic focus compared to the resection margins (Fig. 4B). The histological fibrosis score and the ECM fraction were significantly higher within the fibrotic focus compared to the resection margin. Measures regarding the ECM and collagen structure including the fractal dimension, the alignment and color scores were also higher within the fibrotic focus, accordingly the lacunarity was lower within the fibrotic focus compared to the resection margins (Fig. 4C). Although pairwise comparison showed a significant difference between the samples from the resection margins and the fibrotic focus, we analyzed the differences within both samples from each patient individually. The histological fibrosis score and the fractal dimension were the only parameters able to correctly discriminate the fibrotic focus from the resection margin in each patient (positive difference Δ for tissue pairs from each patient; Fig. 4D).

## Discussion

Our primary aim of this study was to find a new observer-independent quantitative histomorphometric method to measure the degree of intestinal fibrosis in histological samples from surgical specimen of patients with CD based on the structural analysis of the ECM. Our approach was guided by the hypothesis, that during fibrosis development there is not only an accumulation of ECM but also a structural remodeling of this matrix and that these structural changes could be quantified to grade fibrosis severity. We have identified fractal dimension analysis as well as picrosirius polarized light microscopy as tools to quantify the structural changes of the ECM in histological samples of surgical specimens of patients with CD and could demonstrate, that structural changes of the ECM indeed correlate with fibrosis severity as determined by established histological and histomorphometric fibrosis scores.

Fractal analysis of histological samples with the determination of the fractal dimension using the box counting method has already been used to quantify fibrosis severity in other organ systems such as the liver and the heart^23,24^. In these studies, the fractal dimension increases with fibrosis severity, thus indicating, that besides an accumulation of ECM, spatial structural changes of the ECM are present during fibrosis development in these organ systems.

Fractal analysis is a mathematical method to describe the complexity of a pattern or structure that cannot be captured by classical Euclidian geometry. While it is common sense that most lines are one-dimensional objects, surfaces are two-dimensional objects and volumes are three-dimensional, nature exhibits geometrical structures that do not quite fit into this simple hierarchy. Unlike our common understanding of integer-valued dimensions, fractal dimension analysis allows us to assign any positive real number as a dimension to an object. For objects that are not perfect surfaces, but possess many holes, we may want to assign a dimension close to two but slightly less. The idea of non-integer dimensions is due to Hausdorff and dates back to the beginning of the twentieth century. It has been popularized by Mandelbrot in his monograph^25^. In our case, we analyze the fractal dimension of ECM fibers within histological sections that due to their complex geometry exhibit a dimension that lies between 1 and 2. With fractal analysis using the box counting method we may thus assign the same object, here the ECM fibers, a different dimension depending on the complexity of the fiber arrangement as we show to be inherently correlated with the degree of fibrosis. The basic idea to define a fractal dimension by the box counting method is to study a scaling limit: One studies the logarithm of how many boxes of size *L* one needs to cover the object and divides this by the logarithm of 1/*L*. As the box length tends to zero, this yields the fractal dimension. Due to restrictions imposed by pixel sizes, computer algorithms can never take the actual scaling limit of infinitesimal box lengths but stop at a finite box length that is defined by the resolution of the image or as adjusted in the defined algorithm.

The FIJI/ImageJ plugin FracLac makes fractal analysis with the box counting method easily available for digital image analysis^15^. In our study, we used scans of Masson trichrome stained histological sections for fractal analysis and adapted the image preprocessing from Jiang et al. who used fractal analysis to analyze ECM fiber arrangement during wound healing and scar formation^12^. In our study, the fractal dimension positively correlated with the semiquantitative histological fibrosis score and slides with different fibrosis scores showed statistically significant differences regarding the fractal dimension (Fig. 1B). These findings support our hypothesis, that structural changes in the ECM are a feature of intestinal fibrosis and that this can be used to quantify the degree of intestinal fibrosis in histological samples from Crohn’s patients. Interestingly, samples with a fibrosis score of 1 showed a lower fractal dimension and a higher lacunarity than samples with a fibrosis score of 0 indicating an expansion of the ECM network during the initial phase of fibrosis formation. Correa-Gallegos et al. found a similar pattern regarding the structural development of the ECM in a murine wound healing model^10^.

To further characterize the structural changes of the ECM during fibrosis progression, we aimed to selectively observe the collagen network and distinguish it from the entirety of the ECM, by combining picrosirius red staining with polarized light microscopy. Using the picrosirius red staining method in combination with polarized light microscopy allows for the selective visualization of the collagen network in histological sections^21^. We found that in sections with no to little fibrosis, most of the collagen network was arranged in a meshwork like pattern while in samples with advanced fibrosis, the collagen fibers were arranged mostly in thick fibers and bundles. Work by Komuro et al. and Orberg et al. has revealed the structure of the collagen matrix in healthy intestine as a lattice like structure that corresponds to our findings of the meshwork-like pattern seen in picrosirius polarized light microscopy^22,26^. In samples with higher fibrosis score, the pattern changed, and thicker, more prominent collagen fibers became apparent. As the fiber arrangement was heterogeneous within individual histological sections, we developed a score to quantify the fiber arrangement, taking into account this heterogeneity within individual samples (Table 3). The development of an automated digital image analysis tool would here be beneficial to better quantify these findings. However, automated whole slide scanning using polarized light is not widely available limiting the accessibility of this method.

There is much debate about the color of the polarized light that results from picrosirius polarized light microscopy of collagenous tissue. The polarized light color has been suggested to depend on the type of collagen (type I collagen was associated with red polarized light and type III collagen was associated with green polarized light), the collagen fiber thickness and the orientation of the histological section compared to the linear polarization filters^18,27^. In our samples, we found the polarized color to range from orange-yellow to green. Samples with a high fibrosis score showed a green dominance of the collagen fibers while samples with no to minimal fibrosis showed mostly orange-yellow color. Although it has been shown that the polarized light color does not entirely depend on the composition of the collagen fibers regarding type I and III collagen, we performed gene expression analysis on RNA isolated from the corresponding FFPE slides. With increasing histological fibrosis scores, expression of both, *COL1A1* and *COL3A1*, increased while the ratio of *COL1A1/COL3A1* was higher in samples with a high fibrosis score. Immunofluorescence staining was performed to support these finding on a protein expression level. This underlines, that the color of the polarized light is not dependent on the type of collagen within the collagen fibers. Conclusions about the exact mechanism that contributes to color differences in picrosirius polarized light microscopy between histological sections with no and those with advanced fibrosis cannot be drawn from our data. Nevertheless, our findings on differences in collagen fiber alignment and color using picrosirius polarized light microscopy underline the structural changes of the collagen architecture during fibrosis progression.

Summarized, we can conclude the following from our data: (I) Intestinal fibrosis in CD is associated with increasing complexity of the ECM network in histological section, (II) fractal analysis can be used to quantify these structural changes in the ECM network and thus can be used as a quantitative tool for the analysis of intestinal fibrosis that is superior to the analysis of the proportionate ECM fraction, (III) picrosirius polarized light microscopy supports these findings on structural changes of the collagen network during progressing fibrosis and semiquantitative scoring systems have been developed to further analyze these structural changes with regard to the collagen fraction of the ECM, (IV) the *COL1A1/COL3A1* gene expression ratio increases in advanced fibrosis in CD.

Despite recent advances in biological therapy for CD, a high proportion of patients still develop fibrotic intestinal strictures and associated fistulas that require surgical resections^28^. The development of anti-fibrotic therapies requires adequate diagnostic modalities to quantify intestinal fibrosis to allow for evaluation of treatment outcomes of potential therapeutics. As intestinal fibrotic strictures in CD are not accessible for biopsies and histological evaluation without surgical resection, radiographic methods are needed to measure therapeutic successes. These radiological methods are usually validated by histological fibrosis scores of patients undergoing bowel resection surgery for CD with preoperative radiological imaging. However, so far there is no histological scoring systems that has been considered appropriate for clinical trials^8,9^. Furthermore, existing fibrosis scoring systems are observer dependent and are not based on observer-independent automated digital histological image analysis^7^. As demonstrated in this study, structural analysis of the ECM network may be a valuable complement to previously established histological semiquantitative scoring systems for intestinal fibrosis in CD. One major advantage compared to established scores is, that is it observer independent. Furthermore, the methods described in our study could be applied to preclinical models for the study of intestinal fibrosis and has the potential to distinguish between different fibrosis degrees more precisely than semiquantitative fibrosis scores.

However, this study has some limitations. Firstly, we only performed our methods on one dataset and although image preprocessing prior to fractal analysis was performed to limit the effect of differences in staining intensity on the results, differences in tissue preprocessing and fabrication of FFPE slides could still influence the results. Secondly, we only scored the structural differences of the collagen network visualized by picrosirius polarized light microscopy using semiquantitative scores. As we did not have a polarized light slide scanner available, digital image analysis of whole scans was not possible. We focused on manual scoring during microscopy due to the heterogeneity within individual histological sections. Thus, the results from picrosirius polarized light microscopy only underline our findings on structural changes of the ECM network during fibrosis progression but have limited practicability for automated quantification so far. Furthermore, it must be acknowledged that this study has a limited sample size of 28 patients with two histological samples per patient. However, despite the small sample size, our findings not only provide a potential new tool to quantify and compare intestinal fibrosis in histological samples of patients with CD but also give new insights into changes of the ECM structure during fibrosis progression. The findings of our study are supported by previous studies of other authors that could demonstrate the correlation of fibrosis progression in the liver and heart with structural changes of the ECM as quantified by fractal analysis^23,24,29^. Regarding the generalizability of the method to new data sets, it should be noted that some variability in histological slide preparation and staining (thickness of histological sections, staining properties of individual staining kits) may influence the histological analysis. When applying the methods described in this study to other datasets, we recommend paying special attention to the consistency of histological slide preparation within the dataset. Comparison of true fractal dimension values between datasets may be limited.

Nevertheless, we believe that our results provide new insights into the structural changes of the ECM during fibrosis progression, based on which quantitative analyses for observer-independent measurement of the degree of fibrosis can be performed. As the method of fractal analysis for the quantification of fibrosis has already been validated in other organ systems, we are highly optimistic that our results can be reproduced by other investigators and in other data sets. Measuring the degree of fibrosis in surgical specimens from patients with CD may be an important factor in determining optimal postoperative medical therapy, especially in the context of potential future anti-fibrotic drugs.

### Supplementary Information

Supplementary Information.

## Data Availability

Relevant data underlying this article are available in the article and in its online supplementary material.
