# Supplementary material for: Fractal analysis of extracellular matrix for observer-independent quantification of intestinal fibrosis in Crohn’s disease
Source: Sci Rep. 2024 Feb 17;14:3988. doi: 10.1038/s41598-024-54545-4 (PMC10874456; doi:10.1038/s41598-024-54545-4)
Supplement: Supplementary file 1 — Supplementary Information. [file 41598_2024_54545_MOESM1_ESM.pdf]

Fractal analysis of extracellular matrix as a new histological method for observer-independent quantification of intestinal fibrosis in Crohn's disease

Marie-Christin Weber[1,#]; Konstantin Schmidt[1,#]; Annalisa Buck[1,2]; Atsuko Kasajima[3]; Simon Becker[4]; Chunqiao Li[1], Stefan Reischl[1,5]; Dirk Wilhelm[1]; Katja Steiger[3]; Helmut Friess[1]; Philipp-Alexander Neumann[1,2]

[1] *Department of Surgery, TUM School of Medicine, Technical University of Munich, Germany*

[2] *Institute for Advanced Study, Technical University of Munich, Germany*

[3] *Institute of Pathology, TUM School of Medicine, Technical University of Munich, Germany*

[4] *Department of Mathematics, ETH Zurich, Switzerland*

[5] Department of Diagnostic and Interventional Radiology, TUM School of Medicine, Technical University of Munich, Germany

[#] *equal contribution*

– Supplementary Information –

Supplementary Figures

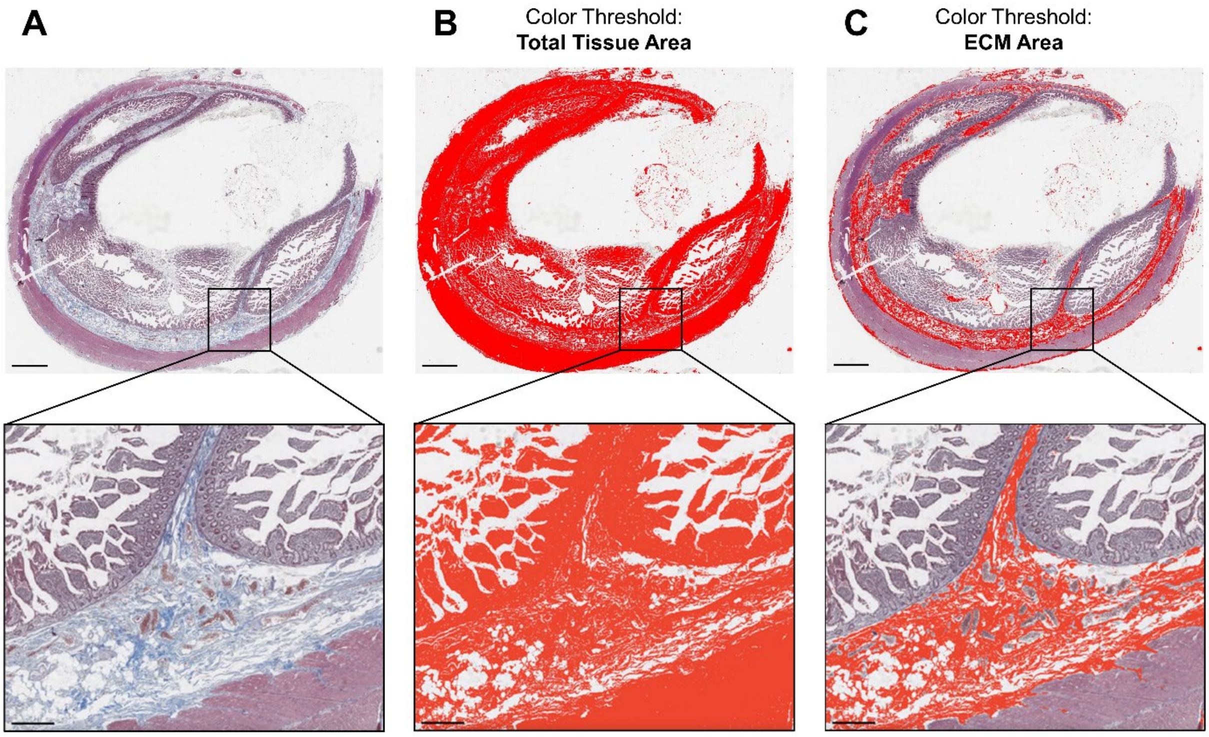

Supplementary Figure 1: Histomorphometric definition of proportionate ECM fraction. *(A) Masson trichrome stained histological sections were scanned and exported to ImageJ as described in the methods section. (B) The total tissue area was determined using a color threshold with the following baseline parameters: Hue 0 - 255, Saturation 4 - 255, Brightness 0 – 232. (C) The collagen/ECM area was determined using a color threshold with the following baseline parameters: Hue 128 - 184, Saturation 4 - 255, Brightness 0 - 232. Threshold cut-offs were checked for appropriateness for each samples and adjusted when needed to tag the correct area. The proportionate ECM fraction was calculated by dividing the collagen area by the total tissue area. Scale bar are 2500µm (upper row) and 500µm (bottom row). ECM = extracellular matrix.*

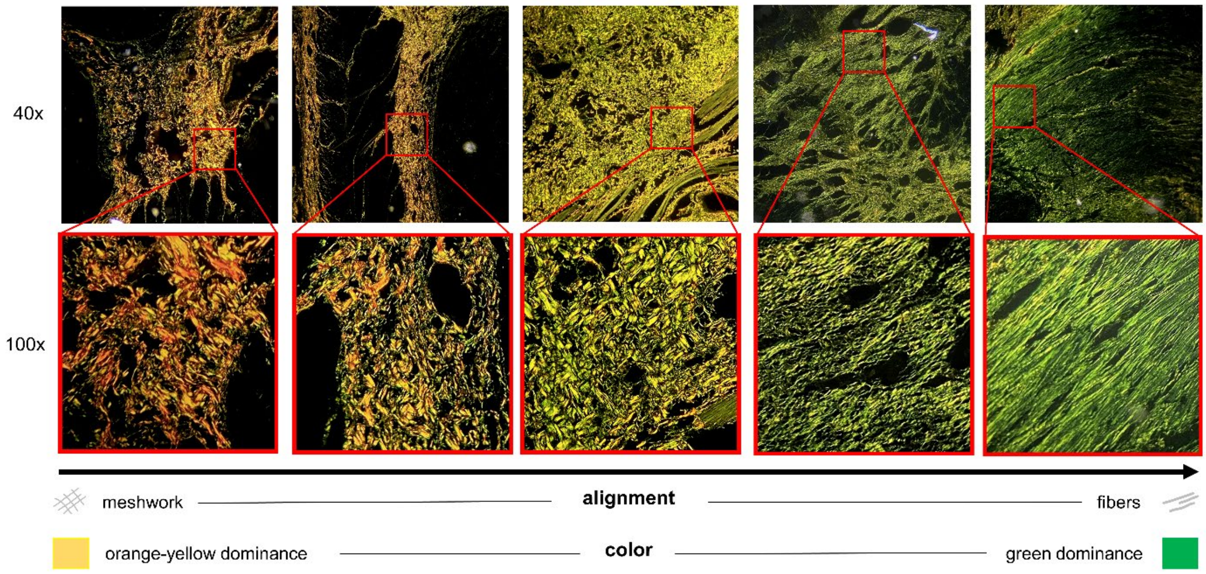

Supplementary Figure 2: Picrosirius polarized light microscopy, representative images. *Two main structural characteristics of fiber alignment were identified, a meshwork-like pattern and parallel arranged fibers. A semiquantitative score ranging from 1 to 5 was applied to quantify fiber alignment (1: meshwork-like pattern, 2: predominant meshwork-like pattern, 3: mixed pattern, 4: predominant parallel fiber alignment, 5: parallel fiber alignment). The color of the fibers in polarized light ranged from orange-yellow to green. A semiquantitative score ranging from 1 to 5 was applied to quantify fiber color (1: orange-yellow dominant, 2: predominant orange-yellow fibers with occasional green fibers, 3: mixed yellow and green fibers, 3: mixed yellow and green fibers, 4: predominant green fibers with occasional orange-yellow fibers, 5: green dominant).*

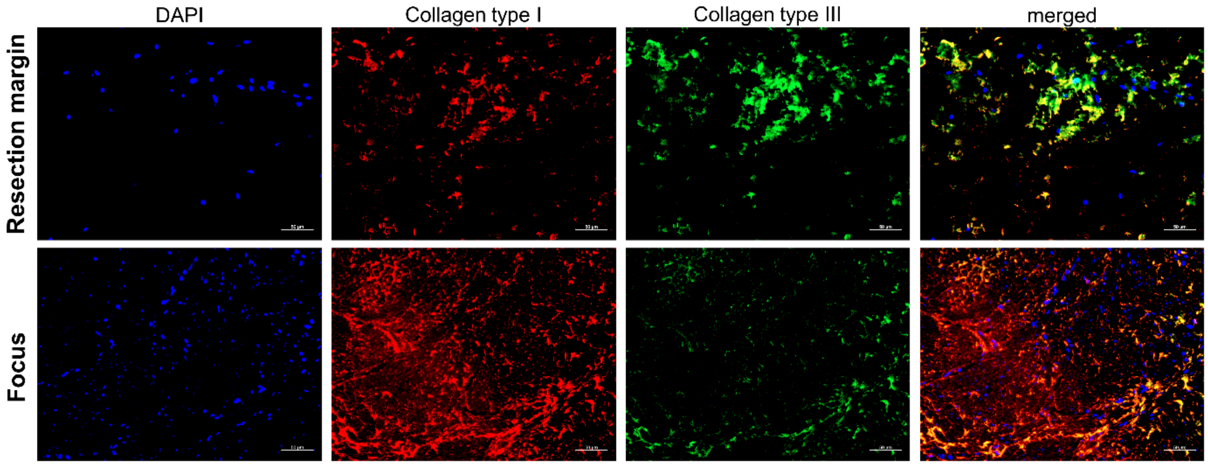

Supplementary Figure 3: Representative immunofluorescence images for Collagen type I and Collagen type III. *Representative images of immunofluorescence staining for Collagen type I (red) and Collagen type III (green) in histological sections from the resection margin (upper row) and the fibrotic focus (bottom row). The focus demonstrates higher cell density due to immune cell infiltrates (see DAPI staining). In the resection margin, collagen fibers seem the contain both types of collagens whereas in the fibrotic focus, collagen type I is dominant. Scale bar is 50µm.*

SUPPLEMENTARY MATERIAL 4: DETAILS ON FRACTAL ANALYSIS

1. What is the box-counting dimension?

Let us consider a binary image by which we mean a planar domain Ω, e.g. a square, such that every point $x \in \Omega$ is assigned a value 0 (black) or 1 (white). A binary image is therefore nothing else as a function $f : \Omega \to \{0, 1\}$ such that $f(x)$ is the color code.

One way to define the dimension of the white region of an image is to use the concept of *fractal dimension* or *box-counting dimension*, see [30] for applications to biology. We cover the white region of an image by boxes of side length ε. Indeed, there are many ways to cover a region with boxes and thus we define $N(\varepsilon)$ the minimal number of such boxes required.

The fractal dimension is then defined as the limit as our box-size parameter ε tends to zero

$$\dim(\Omega) = \lim_{\varepsilon \downarrow 0} \frac{\log(N(\varepsilon))}{\log(1/\varepsilon)}.$$

To numerically analyze the fractal dimension of our binary images, we used the FIJI/ImageJ plugin FracLac, developed by A. Karperien.

We shall now try to illustrate this concept by studying a few examples.

2. Fractal dimension does not depend on mass

Let us consider the following two binary images in Figure 1.

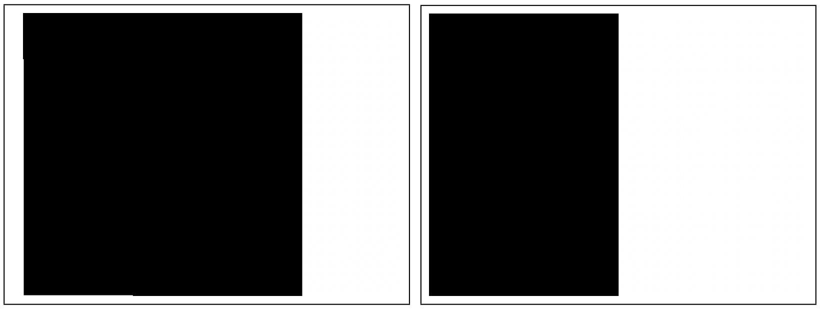

Figure 1. Two binary images of same fractal dimension 2, even though the area of the two white areas is different.

To determine fractal dimension, we proceed as explained before. We may identify the domain of each image with the unit square $\Omega = [0, 1]^2$.

Then, we have to fix $\varepsilon > 0$ and ask ourselves how many squares fit into the white part of each image, respectively. Into the entire domain $[0, 1]^2$ we could fit approximately $1/\varepsilon^2$

many squares, since each such square has a volume ε^2 and the volume of the full square is one.

Hence, since roughly 1/4 of the full square is white on the picture on left and 1/2 of the full square on the picture on the right, we find

$$N_{\leftarrow}(\varepsilon) \approx \frac{1}{4\varepsilon^2} \text{ and } N_{\rightarrow}(\varepsilon) \approx \frac{1}{2\varepsilon^2}.$$

Inserting this into into the definition of the fractal dimension, we find for the image on the left

$$\dim(\Omega_{\leftarrow}) = \lim_{\varepsilon \downarrow 0} \frac{\log(N_{\leftarrow}(\varepsilon))}{\log(1/\varepsilon)} \approx \lim_{\varepsilon \downarrow 0} \frac{\log(\frac{1}{4\varepsilon^2})}{\log(1/\varepsilon)} = \lim_{\varepsilon \downarrow 0} \frac{\log(\varepsilon^{-2})}{\log(\varepsilon^{-1})} = 2.$$

Similarly, for the image on the right

$$\dim(\Omega_{\rightarrow}) = \lim_{\varepsilon \downarrow 0} \frac{\log(N_{\rightarrow}(\varepsilon))}{\log(1/\varepsilon)} \approx \lim_{\varepsilon \downarrow 0} \frac{\log(\frac{1}{2\varepsilon^2})}{\log(1/\varepsilon)} = \lim_{\varepsilon \downarrow 0} \frac{\log(\varepsilon^{-2})}{\log(\varepsilon^{-1})} = 2.$$

Since the answer in either case is 2, we see that the fractal dimension does not depend on the volume of the white mass. In fact, it only tells us that in either case the white mass is a two-dimensional object.

3. Generating images of arbitrary fractal dimension

A mathematical way of generating images of arbitrary fractal dimension in the interval [1, 2] is to use fractal Brownian motion (fBm) first introduced in [25]. Fractal Brownian motion is a Gaussian process characterized by a parameter $H \in (0, 1)$ called the *Hurst parameter*. It can be proven that the graph of fractal Brownian motion with Hurst parameter H has fractal dimension $2 - H$. The graph of two different fBms is illustrated in Figure 2.

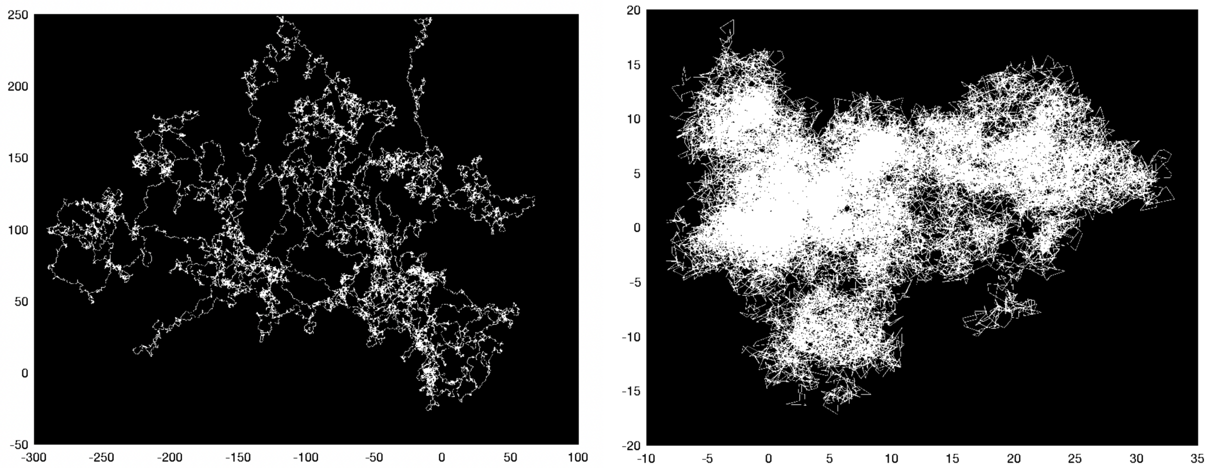

Figure 2. fBm with Hurst parameter $H = 0.7$ on the left and with $H = 0.3$ on the right with same runtime. We emphasize the different scaling of the axes.

References

1. D'Alessio S, Ungaro F, Noviello D, Lovisa S, Peyrin-Biroulet L, Danese S. Revisiting fibrosis in inflammatory bowel disease: the gut thickens. Nature reviews Gastroenterology & hepatology. 2022;19(3):169-84.
2. Rieder F, Fiocchi C. Intestinal fibrosis in inflammatory bowel disease - Current knowledge and future perspectives. J Crohns Colitis. 2008;2(4):279-90.
3. Gordon IO. Histopathology of Intestinal Fibrosis. In: Rieder F, editor. Fibrostenotic Inflammatory Bowel Disease. Cham: Springer International Publishing; 2018. p. 159-71.
4. Chen W, Lu C, Hirota C, Iacucci M, Ghosh S, Gui X. Smooth Muscle Hyperplasia/Hypertrophy is the Most Prominent Histological Change in Crohn's Fibrostenosing Bowel Strictures: A Semiquantitative Analysis by Using a Novel Histological Grading Scheme. J Crohns Colitis. 2017;11(1):92-104.
5. Thia KT, Sandborn WJ, Harmsen WS, Zinsmeister AR, Loftus EV, Jr. Risk factors associated with progression to intestinal complications of Crohn's disease in a population-based cohort. Gastroenterology. 2010;139(4):1147-55.
6. Roda G, Chien Ng S, Kotze PG, Argollo M, Panaccione R, Spinelli A, et al. Crohn's disease. Nature reviews Disease primers. 2020;6(1):22.
7. Gordon IO, Bettenworth D, Bokemeyer A, Srivastava A, Rosty C, de Hertogh G, et al. Histopathology Scoring Systems of Stenosis Associated With Small Bowel Crohn's Disease: A Systematic Review. Gastroenterology. 2020;158(1):137-50.e1.
8. Gordon IO, Bettenworth D, Bokemeyer A, Srivastava A, Rosty C, de Hertogh G, et al. International consensus to standardise histopathological scoring for small bowel strictures in Crohn's disease. Gut. 2022;71(3):479-86.
9. De Voogd FA, Mookhoek A, Gecse KB, De Hertogh G, Bemelman WA, Buskens CJ, et al. Systematic Review: Histological Scoring of Strictures in Crohn's Disease. Journal of Crohn's and Colitis. 2020;14(6):734-42.
10. Correa-Gallegos D, Jiang D, Christ S, Ramesh P, Ye H, Wannemacher J, et al. Patch repair of deep wounds by mobilized fascia. Nature. 2019;576(7786):287-92.
11. Cross SS. Fractals in pathology. The Journal of pathology. 1997;182(1):1-8.
12. Jiang D, Correa-Gallegos D, Christ S, Stefanska A, Liu J, Ramesh P, et al. Two succeeding fibroblastic lineages drive dermal development and the transition from regeneration to scarring. Nat Cell Biol. 2018;20(4):422-31.
13. Bankhead P, Loughrey MB, Fernández JA, Dombrowski Y, McArt DG, Dunne PD, et al. QuPath: Open source software for digital pathology image analysis. Sci Rep. 2017;7(1):16878.
14. Schindelin J, Arganda-Carreras I, Frise E, Kaynig V, Longair M, Pietzsch T, et al. Fiji: an open-source platform for biological-image analysis. Nat Methods. 2012;9(7):676-82.
15. Karperien A. FracLac for ImageJ 1999-2013 [Available from: http://rsb.info.nih.gov/ij/plugins/fraclac/FLHelp/Introduction.htm.
16. Adler J, Punglia DR, Dillman JR, Polydorides AD, Dave M, Al-Hawary MM, et al. Computed tomography enterography findings correlate with tissue inflammation, not fibrosis in resected small bowel Crohn's disease. Inflammatory bowel diseases. 2012;18(5):849-56.
17. Li XH, Fang ZN, Guan TM, Lin JJ, Sun CH, Huang SY, et al. A novel collagen area fraction index to quantitatively assess bowel fibrosis in patients with Crohn's disease. BMC gastroenterology. 2019;19(1):180.

18. Junqueira LC, Cossermelli W, Brentani R. Differential staining of collagens type I, II and III by Sirius Red and polarization microscopy. Arch Histol Jpn. 1978;41(3):267-74.

19. Courtoy GE, Leclercq I, Froidure A, Schiano G, Morelle J, Devuyst O, et al. Digital Image Analysis of Picrosirius Red Staining: A Robust Method for Multi-Organ Fibrosis Quantification and Characterization. Biomolecules. 2020;10(11).

20. Testa LC, Jule Y, Lundh L, Bertotti K, Merideth MA, O'Brien KJ, et al. Automated Digital Quantification of Pulmonary Fibrosis in Human Histopathology Specimens. Frontiers in medicine. 2021;8:607720.

21. Rittié L. Method for Picrosirius Red-Polarization Detection of Collagen Fibers in Tissue Sections. Methods Mol Biol. 2017;1627:395-407.

22. Komuro T. The lattice arrangement of the collagen fibres in the submucosa of the rat small intestine: scanning electron microscopy. Cell Tissue Res. 1988;251(1):117-21.

23. Moal F, Chappard D, Wang J, Vuillemin E, Michalak-Provost S, Rousselet MC, et al. Fractal dimension can distinguish models and pharmacologic changes in liver fibrosis in rats. Hepatology (Baltimore, Md). 2002;36(4 Pt 1):840-9.

24. Zouein FA, Kurdi M, Booz GW, Fuseler JW. Applying fractal dimension and image analysis to quantify fibrotic collagen deposition and organization in the normal and hypertensive heart. Microscopy and microanalysis : the official journal of Microscopy Society of America, Microbeam Analysis Society, Microscopical Society of Canada. 2014;20(4):1134-44.

25. Mandelbrot BB. The Fractal Geometry of Nature: Henry Holt and Company; 1983.

26. Orberg J, Baer E, Hiltner A. Organization of collagen fibers in the intestine. Connect Tissue Res. 1983;11(4):285-97.

27. Lattouf R, Younes R, Lutomski D, Naaman N, Godeau G, Senni K, et al. Picrosirius red staining: a useful tool to appraise collagen networks in normal and pathological tissues. J Histochem Cytochem. 2014;62(10):751-8.

28. Stöss C, Berlet M, Reischl S, Nitsche U, Weber MC, Friess H, et al. Crohn's disease: a population-based study of surgery in the age of biological therapy. International journal of colorectal disease. 2021;36(11):2419-26.

29. Dioguardi N, Grizzi F, Bossi P, Roncalli M. Fractal and spectral dimension analysis of liver fibrosis in needle biopsy specimens. Anal Quant Cytol Histol. 1999;21(3):262-6.

30. Mandelbrot BB, Ness JWV. Fractional Brownian Motions, Fractional Noises and Applications. SIAM Review. 1968;10(4):422-37.
